# *Notes from the Field*: Surveillance for Multisystem Inflammatory Syndrome in Children — United States, 2023

**DOI:** 10.15585/mmwr.mm7310a2

**Published:** 2024-03-14

**Authors:** Anna R. Yousaf, Katherine N. Lindsey, Michael J. Wu, Ami B. Shah, Rebecca J. Free, Regina M. Simeone, Laura D. Zambrano, Angela P. Campbell, MIS-C Surveillance Authorship Group, Steven Crook, Amy Clark, Tiffanie Fulton-Kennedy, Ashley Gent, Walaa Elbedewy, Gabrielle Williams, Amanda Hartley, Kaleb Kitchens, Gillian Richardson, Marion Deming, Cole Burkholder, Jacob Reece, Tom Haupt, Amanda Mandi, Paige D’Heilly, Ayotola Falodun, C.J. Gil, Chelsea Campbell, Kimberly Carlson, Heather D. Reid, Deepam Thomas, Haytham Safi, Jacqueline Denning, Stacy Davidson, Maya Scullin, Allison Longenberger, Kelly Blythe, Xandy Peterson Pompa, Augustina Manuzak, Spencer Cunningham, Kate Cleavinger, Jannifer Anderson, Carmen Rodriguez, Lesley Roush

**Affiliations:** 1 Coronavirus and Other Respiratory Viruses Division, National Center for Immunization and Respiratory Diseases, CDC.; California Department of Health; California Department of Health; Florida Department of Health; Florida Department of Health; Georgia Department of Public Health; Georgia Department of Public Health; Tennessee Department of Health; Tennessee Department of Health; Louisiana Department of Health; Louisiana Department of Health; Michigan Department of Health & Human Services; Michigan Department of Health & Human Services; Wisconsin Department of Health Services; Iowa Department of Health & Human Services; Minnesota Department of Health; North Carolina Department of Health and Human Services; New York State Department of Health; South Carolina Department of Health and Environmental Control; Washington State Department of Health; Illinois Department of Public Health; New Jersey Department of Health; Arkansas Department of Health; Colorado Department of Public Health and Environment; Kentucky Department for Public Health; Ohio Department of Health; Pennsylvania Department of Health; Virginia Department of Health; Arizona Department of Health Services; Hawaii Department of Health; Massachusetts Department of Public Health; Missouri Department of Health and Senior Services; Mississippi State Department of Health; Puerto Rico Department of Health; West Virginia Department of Health.

**Summary**

**What is already known about this topic?**

Multisystem inflammatory syndrome in children (MIS-C) is a rare but serious condition typically occurring 2–6 weeks after SARS-CoV-2 infection and characterized by fever and multiorgan involvement.

**What is added by this report?**

MIS-C incidence has decreased from early in the COVID-19 pandemic (highest in late 2020–early 2021), but cases continue to occur with a recent relative increase in the fall of 2023 after a period of increased COVID-19 activity in the general population. Among 117 patients with MIS-C in 2023, approximately one half required intensive care unit–level care. More than 80% (92 of 112) of MIS-C cases were in vaccine-eligible but unvaccinated children, and among the 20 vaccinated children, 60% likely had waned immunity at the time of MIS-C illness.

**What are the implications for public health practice?**

MIS-C cases continue to occur but at low rates, making ongoing surveillance valuable. COVID-19 vaccination remains important for preventing MIS-C.

Multisystem inflammatory syndrome in children (MIS-C) is a rare but serious condition typically occurring 2–6 weeks after SARS-CoV-2 infection and characterized by fever and multiorgan involvement (*1*,*2*). In May 2020, CDC created an MIS-C case definition and established a passive national surveillance system for voluntary case reporting by state and local health departments.* In 2022, CDC and the Council of State and Territorial Epidemiologists (CSTE) created a new surveillance case definition that went into effect on January 1, 2023^†^ (*3*). Approximately 87% of cases reported using the 2020 case definition also meet the 2023 case definition. This report describes 2023 MIS-C cases and compares them with cases reported earlier in the COVID-19 pandemic.

## Investigation and Outcomes

All MIS-C cases reported to CDC national surveillance as of February 26, 2024, with illness onset during 2023 were included, and patient characteristics were analyzed. Incidence (cases per 1,000,000 person-months) was estimated using bridged-race 2020 population estimates from U.S. Census Bureau data (*4*). COVID-19 vaccination status was reported for children who were age-eligible for vaccination^§^ at the time of MIS-C illness onset. This activity was reviewed by CDC, deemed not research, and was conducted consistent with applicable federal law and CDC policy.^¶^

Among 117 MIS-C patients with illness onset in 2023, 31 (26%) had onset during August–October, after an increase in COVID-19 activity earlier in the summer; this finding represented a two-thirds increase in case counts compared with the 19 (16%) cases reported with onset during the preceding 3 months.** Overall MIS-C incidence in 2023 was 0.11 cases per million person-months (95% CI = 0.10–0.14), representing an 80% decline in incidence compared with that during April–December 2022 (0.56 cases per million person-months; 95% CI = 0.51–0.62), and a 98% decrease from the peak of 6.79 (95% CI = 6.56–7.03) early in the COVID-19 pandemic (October 2020–April 2021).^††^

The median age of MIS-C patients with illness onset in 2023 was 7 years (Table), whereas the median age during February 2020–January 2022 was 9 years, and during April–December 2022 was 5 years^§§^ (*1*,*2*). A similar decline in MIS-C incidence and shift to a younger age group in 2022 was reported in England (*5*).

**TABLE:** Characteristics of patients with multisystem inflammatory syndrome in children reported to CDC (N = 117)* — United States, 2023

Characteristic	No. (%)
**Age, yrs, median (IQR)**	6.9 (3.4–11.5)
**Age group, yrs**	
<1	8 (6.8)
1–4	34 (29.1)
5–11	50 (42.7)
12–15	17 (14.5)
16–20	8 (6.8)
**Male sex**	72 (61.5)
**Race and ethnicity^†^**	
Asian, non-Hispanic	8 (6.8)
Black or African American, non-Hispanic	30 (25.6)
White, non-Hispanic	40 (34.2)
Hispanic or Latino	27 (23.1)
Other race^§^	7 (6.0)
**U.S. Census Bureau region**	
Northeast	11 (9.4)
Midwest	25 (21.4)
South	55 (47.0)
West	26 (22.2)
**Underlying medical conditions^¶^**	
None	68 (58.1)
Obesity	32 (27.4)
Chronic lung disease (including asthma)	16 (13.7)
Neurologic or neuromuscular or developmental condition	9 (7.7)
Cardiovascular condition	6 (5.1)
Immunosuppressive disorder or malignancy	2 (1.7)
**SARS-CoV-2 testing**	
Positive antibody test result (among 93 patients tested by serology)	88 (94.6)
Positive PCR or antigen test result (among 88 patients tested by PCR or antigen)	27 (30.7)
**Organ system involvement**	
Cardiac**^,††^	63 (53.8)
Elevated troponin	45 (38.5)
Cardiac dysfunction (left or right)	31 (26.5)
Left ventricular ejection fraction <55%	21 (17.9)
Coronary artery dilatation, ectasia, or aneurysm	22 (18.8)
Pericardial effusion or pericarditis	14 (12.0)
Myocarditis	13 (11.1)
Congestive heart failure	2 (1.7)
Shock**^,§§^	40 (34.2)
Hematologic**^,¶¶^	63 (53.8)
Thrombocytopenia (platelets <150,000 cells/*μ*L)	37 (31.6)
Lymphopenia (ALC <1000 cells/*μ*L)	34 (29.1)
Gastrointestinal**^,^***	104 (88.9)
Other abdominal involvement**^†††^**	12 (10.3)
Mesenteric adenitis	3 (2.6)
Appendicitis or inflamed appendix	2 (1.7)
Cholecystitis or inflamed gallbladder	1 (0.9)
Mucocutaneous**^,§§§^	104 (88.9)
**Other symptoms or complications reported**	
Cough	44 (37.6)
Neck pain	26 (22.2)
Shortness of breath	23 (19.7)
Encephalopathy	4 (3.4)
Meningitis or encephalitis	1 (0.9)
**Treatment**	
Intravenous immunoglobulin	100 (85.5)
Steroids	94 (80.3)
**Hospital course and outcomes**	
Oxygen, high flow nasal cannula	20 (17.1)
Invasive mechanical ventilation	13 (11.1)
CPAP or BiPAP	4 (3.4)
Extracorporeal membrane oxygenation	2 (1.7)
No. of days in hospital, median (IQR)	4 (3–7)
ICU-level care^¶¶¶^	58 (49.6)
Death	3 (2.6)
**Reported COVID-19 vaccination status** (among 112 age-eligible**** patients)	
No vaccination	92 (82.1)
Vaccination (any dose) received	20 (17.9)
1 dose received^††††^	4 (3.6)
2 doses received	11 (9.8)
≥3 doses received	5 (4.5)
>12 mos from last vaccine dose to MIS-C onset (among 20 vaccinated patients)	12 (60.0)
Time from most recent vaccine dose to MIS-C onset, no. of days, median (IQR)	401 (247–511)

**Abbreviations:** ALC = absolute lymphocyte count; BiPAP = bilevel positive airway pressure; CPAP = continuous positive airway pressure; CSTE = Council of State and Territorial Epidemiologists; ICU = intensive care unit; MIS-C = multisystem inflammatory syndrome in children; PCR = polymerase chain reaction.

* https://www.cdc.gov/mis/index.html

^†^ Five patients were reported as race or ethnicity unknown or refused to answer.

^§^ Patients defined as Other race were those who identified as non-Hispanic multiple race (one), Native Hawaiian or other Pacific Islander (two), or non-Hispanic other (four).

^¶^ No children with diabetes mellitus or sickle cell disease were reported; obesity was ascertained by clinician diagnosis of obesity or body mass index–based obesity (calculated only in children aged >2 years).

** Per 2023 CSTE/CDC MIS-C surveillance case definition. https://www.cdc.gov/mis/mis-c/hcp_cstecdc/index.html

^††^ Cardiac involvement indicated by left ventricular ejection fraction <55%; coronary artery dilatation, aneurysm, or ectasia; or troponin elevated above normal laboratory range, or indicated as elevated in a clinical note.

^§§^ Shock indicated in a clinical note or receipt of vasopressors.

^¶¶^ Platelet count <150,000 cells/*μ*L or ALC <1,000 cells/*μ*L.

*** Abdominal pain, vomiting, or diarrhea.

^†††^ Other abdominal involvement was defined as having colitis or enteritis, hepatomegaly or splenomegaly, liver failure, intussusception, or free fluid.

^§§§^ Rash, inflammation of the oral mucosa, conjunctivitis or conjunctival injection, or extremity findings (erythema [redness] or edema [swelling] of the hands or feet).

^¶¶¶^ ICU-level care was defined as having a documented ICU admission or having received ICU-level care, including mechanical ventilation, vasopressor support, or extracorporeal membranous oxygenation.

**** A total of 112 patients were age-eligible for vaccination at the time of illness onset. Eight months was considered the minimum age at which a child could plausibly have received 2 doses of a primary mRNA vaccination series, with 6 months being the earliest possible age at first dose and ≤4 weeks from first dose required to complete a 2-dose primary series, and 28 days between time since last dose and hospitalization.

^†††† ^One age-eligible patient was reported to have received 2 COVID-19 vaccine doses; however, the second dose was received <14 days before onset of MIS-C; therefore, the child was categorized as having received only 1 dose before illness onset.

Among the 117 MIS-C patients with illness onset in 2023, 68 (58%) had no underlying medical conditions; 58 (50%) required intensive care unit (ICU)-level care, 40 (34%) experienced shock, and 31 (27%) experienced cardiac dysfunction. These prevalences are similar to published national MIS-C surveillance data for 2,116 cases reported during July 9, 2021–January 31, 2022 (52% requiring ICU-level care, 38% with shock, and 29% with cardiac dysfunction), and are improved compared with data for cases reported for the total 4,470 cases during the earliest part of the pandemic, from February 19, 2020–July 31, 2021 (63% requiring ICU-level care, 45% with shock, and 31% with cardiac dysfunction) (*1*,*2*).

Three (3%) patients with MIS-C died in 2023. Although 112 (96%) patients were age-eligible for COVID-19 vaccination, only 20 (18%) had documented receipt of any COVID-19 vaccine. Among the 48 vaccine-eligible patients with underlying medical conditions, nine (19%) had documented receipt of any COVID-19 vaccine. Among the 20 patients who had received COVID-19 vaccination, 12 (60%) received their last dose >12 months before MIS-C onset.

## Conclusions and Recommendations

MIS-C continues to occur, but at low rates compared with those observed early in the COVID-19 pandemic. MIS-C incidence has declined, a recent shift to cases in younger children has occurred, and clinical characteristics have evolved. The reported 2023 incidence is likely an underestimate because jurisdictional reporting of MIS-C cases with illness onset in 2023 is incomplete, and case counts and incidence might also be affected by the change in case definition that occurred that year. Changes might also reflect changing SARS-CoV-2 population immunity from vaccination and previous infection, and characteristics of the predominant circulating SARS-CoV-2 variants. Clinicians should recognize that MIS-C might occur, especially during and after periods of increased COVID-19 activity, and should be familiar with treatment guidelines.^¶¶^ Continued reporting of MIS-C cases to jurisdictional health departments is important to monitor trends and patients’ demographic and clinical characteristics. MIS-C patients with illness onset in 2023 were predominantly unvaccinated children and those whose vaccine-induced immunity had likely waned. COVID-19 vaccination remains an important tool for preventing MIS-C. CDC recommends that all children aged ≥6 months stay up to date with COVID-19 vaccination to protect against serious COVID-19 illness and complications, including MIS-C.

## Footnotes

*
https://www.cdc.gov/mis/index.html

† The 2023 CSTE/CDC MIS-C case definition differs from the 2020 CDC case definition in the following conditions: 1) no requirement for duration of fever; 2) a C-reactive protein test result of 3.0 mg/dL is required to indicate systemic inflammation; 3) respiratory, renal, and neurologic systems are excluded from organ involvement criteria; 4) shock is added as a separate organ system manifestation; and 5) SARS-CoV-2 testing now includes time parameters (i.e., SARS-CoV-2 viral testing within 60 days of MIS-C hospitalization or serology test during MIS-C illness).

§ For this analysis, 8 months was considered the minimum age by which a child could plausibly have completed an mRNA primary vaccination series, with 6 months being the earliest possible age at first dose, and ≤4 weeks from first dose required to complete the 2-dose primary series, and 28 days between time since last dose and hospitalization.

¶ 45 C.F.R. part 46, 21 C.F.R. part 56; 42 U.S.C. Sect. 241(d); 5 U.S.C. Sect. 552a; 44 U.S.C. Sect. 3501 et seq.

** https://covid.cdc.gov/covid-data-tracker/#mis-national-surveillance (Accessed March 11, 2024).

†† SARS-CoV-2 surveillance data were used to define variant-predominant periods, allowing for 2 weeks to MIS-C onset from when a variant exceeded 50% circulating lineages. Variant-predominant period (dates), number of MIS-C cases, and incidence (cases per 1,000,000 person-months with 95% CI) were defined as follows: pre-Delta (October 15, 2020–April 5, 2021): n = 3,284, incidence = 6.79; 95% CI = 6.56–7.03; Delta (July 10–December 24, 2021): n = 2,300, incidence = 4.90; 95% CI = 4.70–5.10; Omicron BA.1/BA 1.1 (January 1–April 8, 2022): n = 1,149, incidence = 4.21; 95% CI = 3.98–4.46; Omicron BA.2/BA.4/BA.5 (April 9–December 31, 2022): n = 422, incidence = 0.56; 95% CI = 0.51–0.62; and 2023 Omicron subvariants (January 1–December 31, 2023): n = 117, incidence = 0.11; 95% CI = 0.10–0.14.

§§ SARS-CoV-2 surveillance data were used to define variant-predominant periods, allowing for 2 weeks to MIS-C onset from when a variant exceeded 50% circulating lineages. Variant-predominant period (dates) and median age (with IQR) were defined as follows: pre-Delta (October 15, 2020–April 5, 2021): median age = 9.2 years, IQR = 5.4–13.1 years; Delta (July 10–December 24, 2021): median age = 9.1 years, IQR = 5.5–12.3 years; Omicron BA.1/BA 1.1 (January 1–April 8, 2022): median age = 7.5 years, IQR = 4.1–11.5 years; Omicron BA.2/BA.4/BA.5 (April 9–December 31, 2022): median age = 5.4 years, IQR = 2.8–9.8 years; and 2023 Omicron subvariants (January 1–December 31, 2023): median age = 6.9 years; IQR = 3.4–11.5 years.

¶¶
https://www.covid19treatmentguidelines.nih.gov/management/clinical-management-of-children/hospitalized-children-therapeutic-management-of-mis-c/
